# Dental workers in front-line of COVID-19: an *in silico* evaluation targeting their prevention

**DOI:** 10.1590/1678-7757-2020-0678

**Published:** 2021-03-26

**Authors:** Pedro Henrique SETTE-DE-SOUZA, Moan Jéfter Fernandes COSTA, Lucas AMARAL-MACHADO, Fábio Andrey da Costa ARAÚJO, Adauto Trigueiro ALMEIDA, Luiza Rayanna Amorim de LIMA

**Affiliations:** 1 Universidade de Pernambuco Faculdade de Odontologia Programa de Pós-Graduação em Saúde e Desenvolvimento Socioambiental Arcoverde Brasil Universidade de Pernambuco, Faculdade de Odontologia, Programa de Pós-Graduação em Saúde e Desenvolvimento Socioambiental, Arcoverde, Brasil.; 2 Universidade Federal do Rio Grande do Norte Departamento de Odontologia Natal Brasil Universidade Federal do Rio Grande do Norte, Departamento de Odontologia, Natal, Brasil.; 3 Universidade Federal do Rio Grande do Norte Departamento de Farmácia Natal Brasil Universidade Federal do Rio Grande do Norte, Departamento de Farmácia, Natal, Brasil; 4 Universidade de Pernambuco Faculdade de Odontologia Arcoverde Brasil Universidade de Pernambuco, Faculdade de Odontologia, Arcoverde, Brasil.; 5 Universidade de Pernambuco Garanhuns Brasil Universidade de Pernambuco, Bacharelado em Engenharia de Software, Garanhuns, Brasil.; 6 Universidade de Pernambuco Programa de Pós-Graduação em Saúde e Desenvolvimento Socioambiental Laboratório de Biologia Celular e Molecular Garanhuns Brasil Universidade de Pernambuco, Programa de Pós-Graduação em Saúde e Desenvolvimento Socioambiental, Laboratório de Biologia Celular e Molecular, Garanhuns, Brasil.

**Keywords:** Molecular docking simulation, Severe acute respiratory syndrome-related coronavirus, Practice management, Dental, Containment of biohazards

## Abstract

SARS-CoV-2 has high human-human transmission rate. The aerosols and saliva droplets are the main contamination source. Thus, it is crucial to point out that dental practitioners become a high-risk group of contagion by SARS-CoV-2. Based on this, protocols have been recommended to avoid cross-contamination during dental care; however, appropriate evidence has not yet been established.

**Objective:**

Our study sought to make a screening, by *in silico* analysis, of the potential of mouth rinses used in dental practices to prevent the dental workers' contamination by SARS-CoV-2.

**Methodology:**

Multiple sequence comparisons and construction of the phylogenetic tree were conducted using the FASTA code. Therefore, molecular docking investigation between SARS-CoV-2 proteins (Main Protease, Spike Glycoprotein, Non-structure Protein, and Papain-like Protease) and molecules used in dental practices (chlorhexidine digluconate, hydrogen peroxide, cetylpyridinium chloride, povidone-iodine, gallic acid, β-cyclodextrin, catechin, and quercetin) was performed using AutoDock Vina. Moreover, 2D interactions of the complex protein-ligand structure were analyzed by Ligplot+.

**Results:**

The obtained results showed a remarkable affinity between SARS-CoV-2 proteins and all tested compounds. The chlorhexidine digluconate, catechin, and quercetin presented a higher affinity with SARS-CoV-2.

**Conclusions:**

The overall results allowed us to suggest that chlorhexidine is the most suitable active compound in reducing the SARS-CoV-2 salivary load due to its better binding energy. However, *in vivo* studies should be conducted to confirm their clinical use.

## Introduction

The coronavirus disease 2019 (COVID-19) outbreak has drawn attention worldwide since its first identified case in Wuhan – China.^1^ This infectious disease, caused by the Severe Acute Respiratory Syndrome coronavirus (SARS-CoV-2), has spread globally and infected millions of people, leading thousands of individuals to death.^2^ The SARS-CoV-2 has high human-human transmissibility, and the saliva plays an essential role in it. Through the saliva droplets/aerosols inhalation or ingestion from infected people, health people may fall ill.^3,4^ Based on this rationale, asymptomatic patients also are considered a transmission vector, since they are freely carrying out their activities and endangering the population’s health.^5^

Salivary glands and saliva are the main reservoirs to SARS-CoV-2 due to the high-affinity with the host-cell receptor angiotensin-converting enzyme II (ACE2), also found in salivary glands.^6,7^ Then, dental practitioners can be considered a high-risk group of contagion by SARS-CoV-2 due to their exposure to aerosols-generated procedures during dental care.^8,9^ Moreover, a lack of evidence regarding what to do as a pre-procedural protocol to avoid SARS-CoV-2 cross-infection is worrying. It should be resolved to offer more protection to dental workers.

Regarding this issue, some protocols have been recommended worldwide to avoid SARS-CoV-2 dissemination during dental care. Based on this, mouth rinses with hydrogen peroxide, povidone-iodine, or chlorhexidine are used before any dental procedure.^10^ However, once these suggestions have inadequate scientific support,^11^ appropriate evidence must be produced to understand the possible mechanism of action of these compounds. Additionally, clinical studies involving drug testing require time and involve risks for both research groups and researchers.^12^

In this context, *in silico* analyses play a fundamental role in simulating molecular processes to support validation studies between molecular and cellular processes.^13^ Among *in silico* analyses, the docking studies can be emphasized, which evaluate protein-ligand complexes through a series of algorithms to generate scoring functions. Thus, these analyses can predict the biological effects of chemical compounds due to their ability to interact with proteins responsible for the virulence present in the surface.^14^ Therefore, the fastest scenario to test existing drugs on the SARS-CoV-2 proteins (such as Main or Papain-like proteases, Spike glycoprotein, and non-structured proteins) is the *in silico* analysis, which is a robust approach to provide remarkable results. Thus, it could propose initial therapeutic strategies to prevent SARS-CoV-2 contamination by dental workers.^15^

Then, our study aimed at providing preliminary data, using computational tools, of the therapeutic potential of mouth rinses, widely used in dentistry practices, to prevent the contamination of dental workers by SARS-CoV-2 during dental procedures. The absence of interaction between the SARS-CoV-2 proteins and mouth rinses’ compounds was considered the null hypothesis.

## Methodology

### Retrieval of proteins sequences

The crystal structures and FASTA code of SARS-CoV-2 proteins were obtained from the Research Collaboratory for Structural Bioinformatics Protein Data Bank – RCSB PDB (RRID: SCR_012820). Therefore, four different SARS-CoV-2 proteins groups [Main Protease – M^pro^ (6LU7, 6Y2E, 6Y84, 6YB7), Spike glycoprotein (6LVN, 6VSB, 6VXX, 6VYB), Non-structure Protein – NSP (6YHU, 6W4B, 6W4H, 6W37, 6WEY, 6WIQ, 6WIJ), and Papain-like Protease (6W9C)] were selected as molecular targets.

### Sequence alignment, multiple sequence comparisons, and construction of the phylogenetic tree

Multiple sequence comparisons of proteins from SARS-CoV-2 was conducted using a Constraint-based Multiple Alignment Tool (COBALT, RRID: SCR_004152) through the FASTA code, which allowed to construct the phylogenetic tree by using the neighbor-joining method based on the alignment sequences.

### Ligand selection and structure preparation

Eight compounds were selected for *in silico* analyses: five substances commercially used as mouth rinses/mouthwashes [chlorhexidine digluconate – CHX (C_34_H_54_C_l2_N_10_O_14_ – PubChem CID: 9552081), hydrogen peroxide – HP (H_2_O_2_ – PubChem CID: 784), cetylpyridinium chloride – CCP (C_21_H_38_ClN – PubChem CID: 31239), povidone-iodine – PVPI (C_6_H_9_I_2_NO – PubChem CID: 410087)] and three antimicrobial compounds [gallic acid – GA (C_7_H_6_O_5_ – PubChem CID: 370), β-cyclodextrin – BCD (C_42_H_70_O_35_ – PubChem CID: 444041), catechin – CAT (C_15_H_14_O_6_ – PubChem CID: 9064), quercetin – QTN (C_15_H_10_O_7_ – CID: 5280343)].

The 2D structures were retrieved from the National Center for Biotechnology Information (NCBI) chemical structure library (PubChem, RRID: SCR_004284). The files were imported in 2D SDF format and converted to 3D Protein Data Bank format (.pdb) by the Open Babel (RRID: SCR_014920).

The ligand’ rotatable bonds were defined using AutoDock, and the structures were saved as pdbqt files for the use in the docking studies.

### SARS-CoV-2 proteins preparation

The AutoDock (RRID: SCR_012746) was used to delete repeated chains, heteroatoms, and water molecules, add polar hydrogens atoms, and add the charge atoms to the protein structure. Gasteiger charges were computed, and the structure was saved as a pdbqt file for the docking studies.

### Molecular docking procedure

The 3D grids were created by the Autogrid algorithm to generate the grid parameter files (Autogrid, RRID: SCR_015982). Each grid map was set to the center of the Chain A. Docking parameters were set according to the protein (Table 1), and all analyses were conducted with “exhaustiveness = 8”.

**Table 1:** Grid parameters of SARS-CoV-2 proteins

SARS-CoV-2 Proteins	Center			Size		
	X	Y	Z	X	Y	Z
6LU7	-26	13	59	126	126	126
6LVN	10	34	29	46	68	40
6VSB	206	223	227	104	92	126
6VXX	198	223	207	90	102	126
6VYB	197	223	206	126	102	126
6W4B	54	-11	23	56	68	54
6W4H	92	24	23	102	126	92
6W9C	-32	34	26	126	126	126
6W37	-24	14	15	40	40	40
6WEY	-2	5	13	126	90	122
6WIQ	-4	-8	-6	96	100	114
6WJI	8	1	-14	126	100	96
6Y2E	-17	-26	18	126	126	126
6Y84	12	1	5	72	84	90
6YB7	12	1	5	106	126	126
6YHU	-25	25	51	70	100	114

Molecular docking was conducted using AutoDock Vina (RRID: SCR_011958), and the best ligand/protein model was identified based on the binding energy (ΔG – kcal/mol).^16^

### Docking visualization

The results were viewed on UCSF Chimera 1.14 (RRID: SCR_004097). Only one protein from each group was selected for the visualization. The 2D interactions of the complex protein-ligand structure, including hydrogen bonds and the bond lengths, were analyzed by Ligplot+ (RRID: SCR_018249) for the high-affinity bindings.^17^

## Results

### Multiple sequence comparisons and construction of the phylogenetic tree

**Figure 1:**
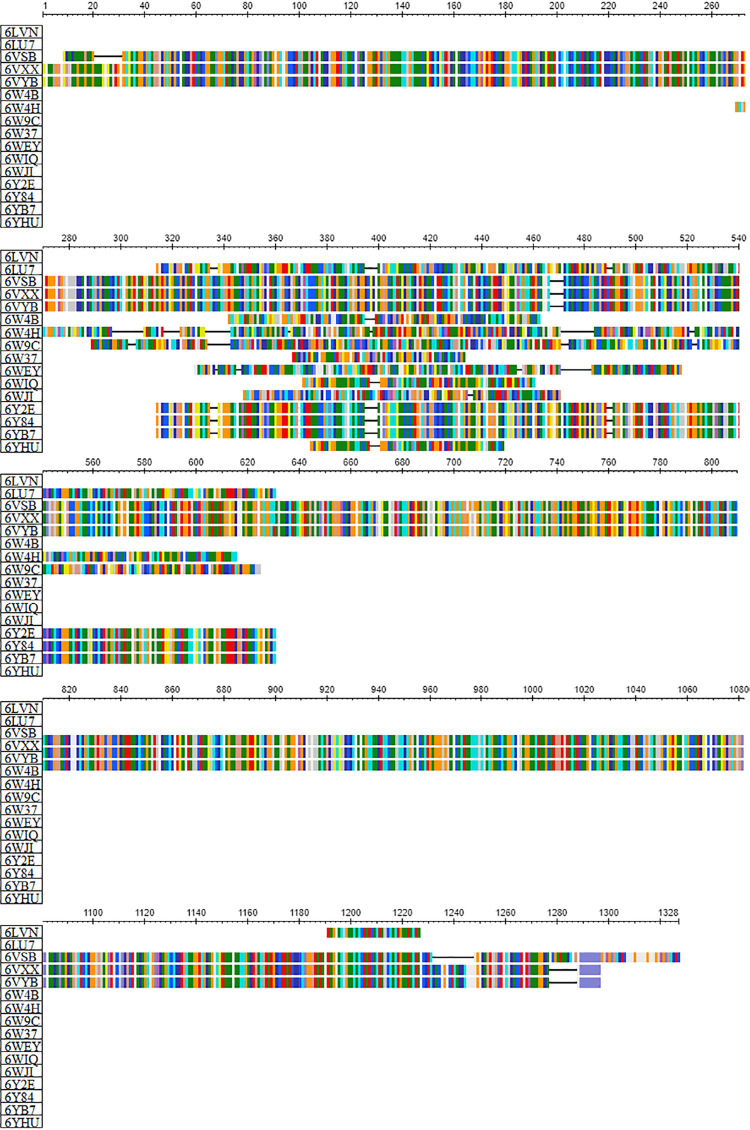
Sequence alignment and multiple sequence comparisons of studied proteins from SARS-CoV-2

**Figure 2:**
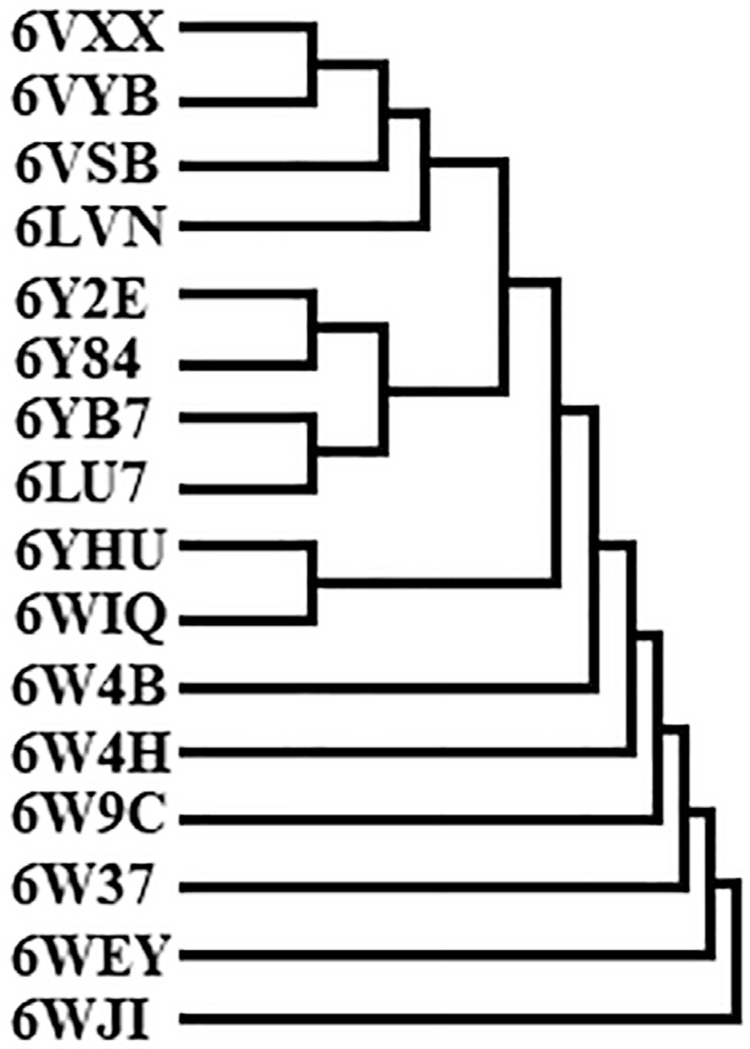
Phylogenetic tree of SARS-CoV-2 studied proteins

Sequence alignment and multiple sequence comparisons of the studied proteins from SARS-CoV-2 (Figure 1) allowed observing their similarity (Figure 2). Moreover, not only the studied M^pro^ presented similarity to the others, but also the Glycoproteins Spike. Besides, two Non-structure Proteins (PDB: 6YHU and 6WIQ) showed similarity, since they presented the same NSP7-NSP8 complex, whereas the other NSP presented several different complexes. In general, the similarity observed in the phylogenetic tree of the studied proteins classes is reflected in the binding energies.

### Molecular docking

**Figure 3:**
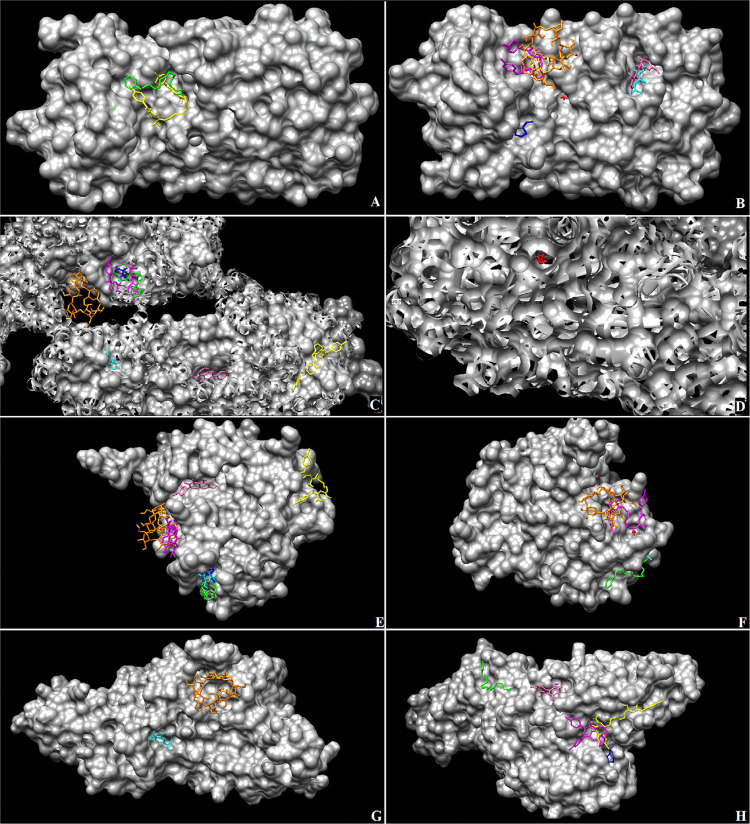
Binding complex and interaction visualization of tested compounds with SARS-CoV-2 proteins. Magenta: chlorhexidine digluconate; Green: cetylpyridinium chloride; Blue: povidone-iodine; Red: hydrogen peroxide; Cyan: gallic acid; Orange: β-cyclodextrin; Yellow: catechin; Pink: quercetin. Main protease - PDB: 6LU7 complex (a, b); Spike Glycoprotein – PDB: 6VYB complex (c, d); Non-structure Protein – PDB: 6W4H complex (e, f); Papain-like Protease – PDB: 6W9C complex (g, h)

The affinity between selected compounds and SARS-CoV-2 proteins was observed in our study (Table 2). Nonetheless, chlorhexidine digluconate, catechin, and quercetin showed higher binding energy than others. A remarkable affinity between chlorhexidine and M^pro^ (PDB: 6Y84, -10.4 kcal/mol) was observed. Hydrogen peroxide showed to be the least recommended compound, since they presented the lowest affinity (-2.1 kcal/mol) with Spike glycoprotein (PBD: 6LVN) and Non-structure Protein (PDB: 6WIQ). The specific binding sites to each compound are shown in Figure 3, in which we could observe that some compounds have the same binding site.

**Table 2:** Binding energy between the tested compounds and the SARS-CoV-2 proteins

SARS-CoV-2 Proteins	Binding Energy (ΔG – kcal/mol)							
	CHX	CCP	PVPI	HP	GA	BCD	CAT	QTN
6LU7	-6.9	-4.2	-3.9	-2.8	-5.5	-5.9	-7.4	-7.5
6LVN	-6.0	-3.6	-2.7	-2.1	-3.8	-4.0	-5.6	-4.8
6VSB	-7.1	-4.6	-4.4	-3.4	-5.0	-5.5	-6.8	-6.4
6VXX	-7.1	-4.5	-3.9	-3.5	-5.5	-6.0	-7.6	-7.2
6VYB	-6.3	-4.3	-3.9	-3.1	-5.3	-5.9	-7.0	-8.5
6W4B	-7.2	-4.3	-3.5	-2.5	-4.7	-4.8	-6.9	-6.7
6W4H	-8.0	-4.9	-4.4	-3.5	-5.9	-7.2	-7.6	-8.6
6W9C	-6.3	-4.7	-4.0	-2.9	-5.2	-5.7	-7.5	-6.6
6W37	-6.6	-3.8	-3.2	-2.5	-4.7	-4.6	-6.1	-6.5
6WEY	-9.6	-4.3	-4.4	-3.4	-5.4	-6.1	-8.0	-8.4
6WIQ	-5.1	-3.6	-3.1	-2.1	-3.6	-4.5	-4.6	-5.8
6WJI	-7.4	-3.5	-3.9	-3.0	-4.8	-6.2	-7.2	-7.4
6Y2E	-5.9	-3.6	-3.8	-2.9	-4.5	-6.1	-6.0	-7.2
6Y84	-10.4	-6.0	-4.5	-3.2	-6.9	-7.9	-9.1	-9.2
6YB7	-9.1	-5.1	-4.2	-3.4	-6.0	-7.1	-8.3	-8.2
6YHU	-5.3	-3.4	-3.4	-2.5	-4.5	-5.0	-4.9	-5.4

CHX: chlorhexidine digluconate; CCP: cetylpyridinium chloride; PVPI: povidone-iodine; HP: hydrogen peroxide; GA: gallic acid; BCD: β-cyclodextrin; CAT: catechin; QTN:

### Interactions of the complex protein-ligand structure

**Figure 4:**
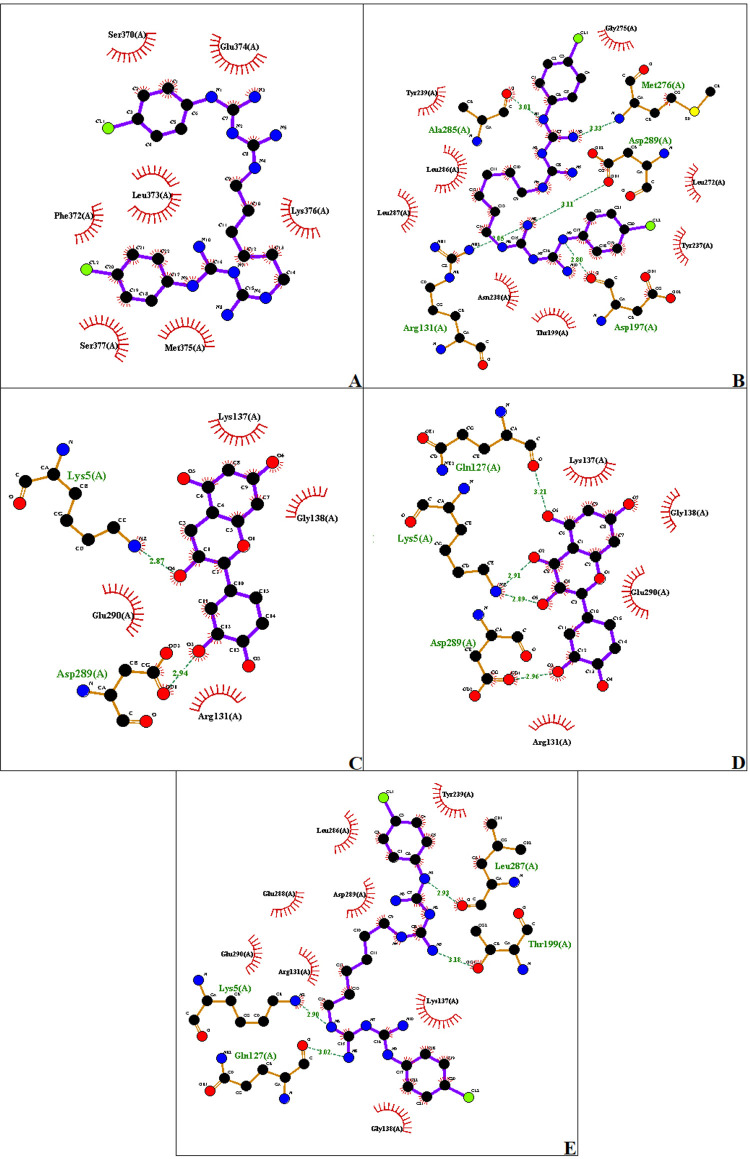
Visualization of residue interactions. Chlorhexidine digluconate-Non structure protein (PDB: 6WEY) binding complex (a); Chlorhexidine digluconate-Mpro (PDB: 6YB7) binding complex (b); Cathechin- Mpro (PDB: 6Y84) binding complex (c); Quercetin- Mpro (PDB: 6Y84) binding complex (d), Chlorhexidine digluconate- Mpro (PDB: 6Y84) binding complex (e)

The results of LigPlot+ analyses showed the interaction of chlorhexidine, catechin, and quercetin with M^pro^ or Non-structure Protein (Figure 4, Table 3). Importantly, these compounds shared the same binding pocket in the M^pro^ (PDB: 6Y84).

**Table 3:** Interaction between the compounds and the protein targets

Protein	Ligand	Interactions
6WEY	CHX	Hydrophobic interaction: Ser370(A), Phe372(A), Leu373(A), Glu374(A), Met375(A), Lys376(A), Ser377(A)
6YB7	CHX	Hydrogen bond: Arg131(A), Asp197(A), Met276(A), Ala285(A), Asp289(A) Hydrophobic interaction: Thr199(A), Tyr237(A), Asn238(A), Tyr239(A), Leu272(A), Gly275(A), Leu286(A), Leu 287(A)
6Y84	CAT	Hydrogen bond: Lys5(A), Asp289(A) Hydrophobic interaction: Arg131(A), Lys137(A), Gly138(A), Glu290(A)
	QTN	Hydrogen bond: Lys5(A), Gln127(A), Asp289(A) Hydrophobic interaction: Arg131(A), Lys137(A), Gly138(A), Glu290(A)
	CHX	Hydrogen bond: Lys5(A), Gln127(A), Thr199(A), Leu287(A) Hydrophobic interaction: Arg131(A), Lys137(A), Gly138(A), Tyr239(A), Leu286(A), Glu288(A), Asp289(A), Glu290(A)

CHX: chlorhexidine digluconate; CAT: catechin; QTN: quercetin.

## Discussion

The null hypothesis was rejected, once the binding affinity among some compounds (chlorhexidine, catechin, and quercetin) and all proteins tested (M^pro^, Spike glycoprotein, Papain-like protease, and Non-structure Protein) was observed.

We constructed the phylogenetic tree of SARS-CoV-2 proteins. We observe that the major proteins in the same group are similar. However, differences in the amino acid residues may affect the interaction between them and “external” molecules. Different affinity degrees were observed besides the similarity among the proteins in the same group. On the other hand, the non-structure protein (NSP) group shows a diversity in the presented complexes, explaining the different sequences and the binding energies among NSP.

The molecular docking showed the affinity of the tested compounds with SARS-CoV-2 proteins in different degrees. In our study, HP and PVPI presented the lowest affinities with SARS-CoV-2 proteins. However, HP and PVPI are reported in the literature as possible products to decrease the SARS-CoV-2 salivary load in dental practice, due to their oxidative propriety.^8,18^ Thus, the obtained data allowed us to hypothesize that their mechanism of action against SARS-CoV-2 would not be binding-dependent. Other studies can also support this hypothesis, demonstrating the anti-SARS-CoV-2 effect of HP and PVPI solutions by *in vitro* studies^19,20^and case report.^21^

On the other hand, CHX was tested against SARS-CoV-2, and the results evidenced better binding energies with all studied M^pro^ and papain-like protease. These SARS-CoV-2 proteases are the main targets of antiviral agents, since they play an essential role in viral RNA replication and controlling host cells.^22^ Thus, our experiments corroborate the data observed by Yoon, et al.^23^ (2020), allowing us to suggest that CHX may avoid the COVID-19 dissemination in the dental office. Additionally, QTN and CAT showed a remarkable affinity to M^pro^, corroborating the previously published studies.^24,25^ All these compounds share the same binding pocket in the M^pro^ (PDB: 6Y84 – Lys5, Arg131, Lys137, Gly138, Asp289, Glu290). Thus, it allows us to suggest their activity against SARS-CoV-2 and possibly develop an effective treatment for COVID-19.

The spike glycoprotein was also studied in our work. The ability to bind to the ACE 2 makes the spike glycoprotein a crucial factor of pathogenicity.^26,27^ Thus, according to Walls, et al.^28^ (2020), this group is an essential target to neutralize SARS-CoV-2. Based on this rationale, the scientific reports describe that the spike glycoprotein in an opened state (PDB: 6VYB) would be associated with the most pathogenic coronaviruses. In contrast, the closed state is associated with a common cold.^28^ In our study, both opened and closed spike glycoprotein had a binding affinity with CHX, CAT, and QTN, showing a possible transmissibility inhibition. However, CHX showed a more expressive binding energy, probably due to the four hydrogen bonds and eight hydrophobic interactions present in its molecule.

Based on our results, we hypothesized that chlorhexidine has two different action mechanisms against SARS-CoV-2: (i) acting on viral RNA replication and controlling host cells; and (ii) neutralizing spike glycoprotein, preventing the binding to the ACE-II. All these mechanisms may decrease pathogenicity in coronaviruses.

Our study revealed the tested compounds’ affinity with SARS-CoV-2 proteins and suggested their effectiveness in preventing virus replication or entering the human cells. Thus, the evidence obtained from molecular docking analysis may guide the development of temporary protocols that can be used to prevent the contamination of dental workers by SARS-CoV-2 during dental procedures in COVID-19 asymptomatic patients.

These findings suggest the possible mechanisms of action of the tested compounds that lead to the susceptibility of SARS-CoV-2. Nonetheless, *in silico* analysis provides preliminary data, which need to be addressed by *in vitro* and/or *in vivo* studies once *in silico* analysis present limitations such as: (i) the evidenced interactions by *in silico* analysis may not mimic the *in vivo* interactions; (ii) the compound-protein interaction may be purely physical, with no clinical significance; (iii) the lack of studies about the anti-SARS-CoV-2 activity of the studied compounds to discuss our findings better. On the other hand, some strengths should be emphasized: (i) it is the first report to suggest the mechanisms for CHX against SARS-CoV-2; and (ii) we conducted a range of analyses to better understand the relationship between SARS-CoV-2 proteins and some compounds used as mouth rinses.

Additionally, it is essential to emphasize that there are no scientific reports regarding effective drugs against SARS-CoV-2. However, our results may provide several usual data in screening useful therapeutic compounds, if further studied by *in vitro* and *in vivo* assays.

## Conclusions

Finally, our findings suggest that chlorhexidine is the most active compound in reducing the SARS-CoV-2 salivary load due to its better binding energy. It can be considered to be used as a mouthwash before dentistry procedures, reducing the SARS-CoV-2 contamination risk of dental workers. However, *in vivo* studies should be conducted to confirm their clinical use.
